# Supplementary material for: Predicting visual function by interpreting a neuronal wiring diagram
Source: Nature. 2024 Oct 2;634(8032):113–23. doi: 10.1038/s41586-024-07953-5 (PMC11446822; doi:10.1038/s41586-024-07953-5)
Supplement: Supplementary file 6 — CRF and ERF predictions for individual TmY4 and TmY9 cells. Analogous to Supplementary Data 3, but for TmY target types. Shown are the top four monosynaptic pathways, the strongest pathway passing through each of the top ten intermediary types (ranking from Extended Data Fig. 7), and the trisynaptic pathway Tm1–TmY–Dm3–TmY (see the section entitled Prediction of spatial normalization). [file 41586_2024_7953_MOESM6_ESM.zip › DataS4/TmY9qΓèÑ/720575940630456375.pdf]

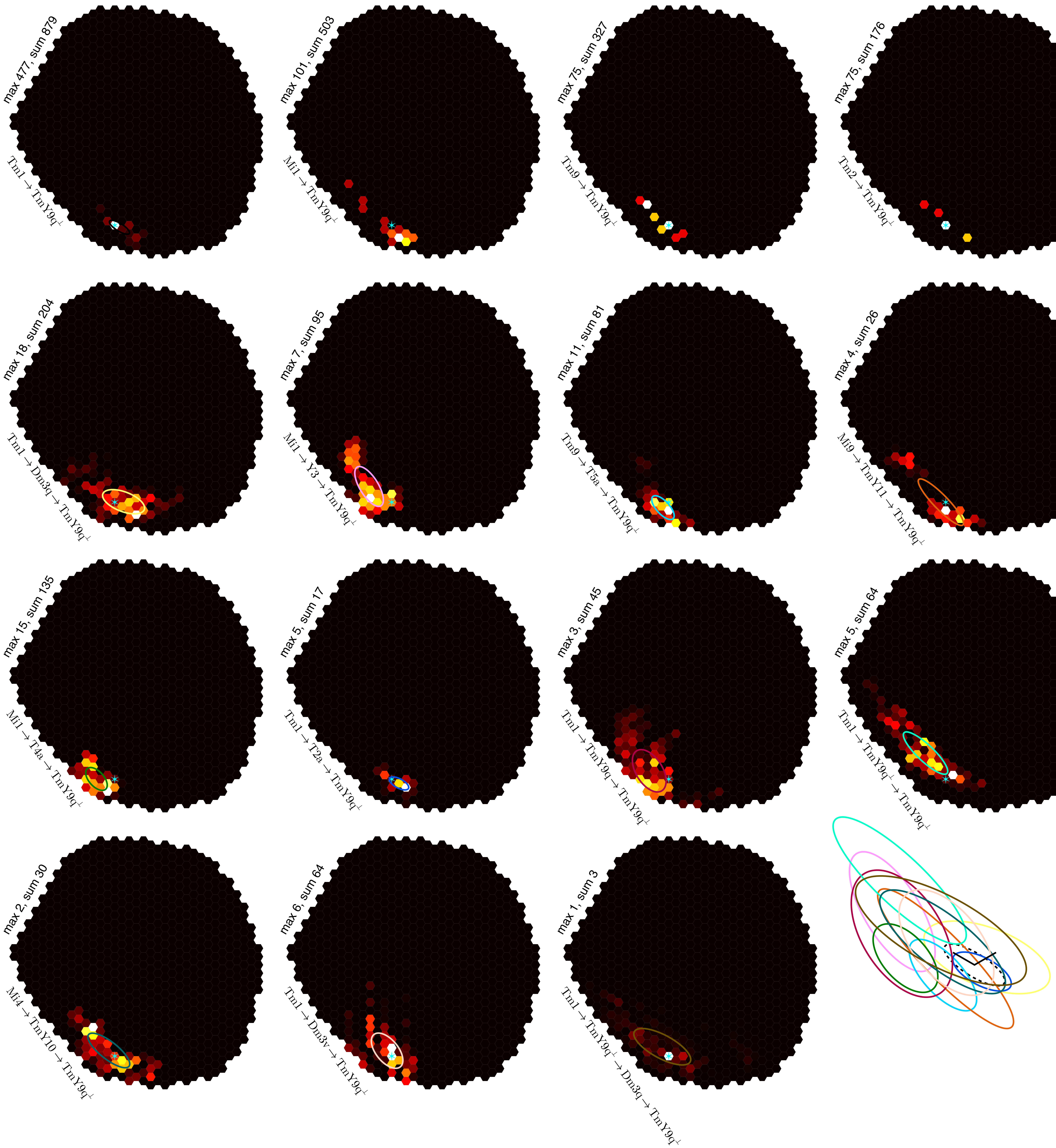
max 477, sum 879
Tm1 → TmY9q⊥
max 101, sum 503
Mi1 → TmY9q⊥
max 75, sum 327
Tm9 → TmY9q⊥
max 75, sum 176
Tm2 → TmY9q⊥
max 18, sum 204
Tm1 → Dm3q → TmY9q⊥
max 7, sum 95
Mi1 → Y3 → TmY9q⊥
max 11, sum 81
Tm9 → T5a → TmY9q⊥
max 4, sum 26
Mi9 → TmY11 → TmY9q⊥
max 15, sum 135
Mi1 → T4a → TmY9q⊥
max 5, sum 17
Tm1 → T2a → TmY9q⊥
max 3, sum 45
Tm1 → TmY9q → TmY9q⊥
max 5, sum 64
Tm1 → TmY9q⊥ → TmY9q⊥
max 2, sum 30
Mi4 → TmY10 → TmY9q⊥
max 6, sum 64
Tm1 → Dm3v → TmY9q⊥
max 1, sum 3
Tm1 → TmY9q⊥ → Dm3q → TmY9q⊥
